# The Properties of High-Performance Concrete with Manganese Slag under Salt Action

**DOI:** 10.3390/ma17071483

**Published:** 2024-03-25

**Authors:** Junchao Yang, Hui Wang, Ling Peng, Fei Zhao

**Affiliations:** 1 School of Civil Engineering, Hebei University of Architecture, Zhangjiakou 075000, China; 2 Hebei Key Laboratory of Diagnosis, Reconstruction and Anti-Disaster of Civil Engineering, Zhangjiakou 075000, China; 3 School of Civil Engineering and Geographic Environment, Ningbo University, Ningbo 315000, China; 4 Department of Mathematics and Physics, Hebei University of Architecture, Zhangjiakou 075000, China

**Keywords:** manganese slag, basalt fibers, mechanical strengths, salt action, X-ray diffraction spectroscopy, scanning electron microscope

## Abstract

Manganese slag (MS) containing a certain amount of active hydration substances may be used as a kind of cementitious material. In the present study, we measured the mass, the relative dynamic modulus of elasticity (RDME), and the flexural and compressive strengths of MS high-performance concrete (MS-HPC) with added basalt fibers exposed to NaCl freeze–thaw cycles (N-FCs), NaCl dry–wet alternations (N-DAs), and Na_2_SO_4_ dry–wet alternations (NS-DAs). Scanning electron microscope energy-dispersive spectrometer (SEM-EDS) spectra, thermogravimetric analysis (TG) curves, and X-ray diffraction spectroscopy (XRD) curves were obtained. The mass ratio of MS ranged from 0% to 40%. The volume ratio of basalt fibers varied from 0% to 2%. We found that, as a result of salt action, the mass loss rate (MLR) exhibited linear functions which were inversely correlated with the mass ratio of MS and the volume ratio of basalt fibers. After salt action, MLR increased by rates of 0~56.3%, but this increase was attenuated by the addition of MS and basalt fibers. Corresponding increases in RDME exhibited a linear function which was positively correlated with MS mass ratios in a range of 0~55.1%. The addition of MS and basalt fibers also led to decreased attenuation of mechanical strength, while the addition of MS led to increased levels of flocculent hydration products and the elements Mn, Mg, and Fe. CaClOH and CaSO_4_ crystals were observed in XRD curves after N-DA and NS-DA actions, respectively. Finally, the addition of MS resulted in increased variation in TG values. However, the opposite result was obtained when dry–wet actions were exerted.

## 1. Introduction

Manganese slag (MS) is a kind of industrial waste produced during metal smelting processes [1,2]. It is estimated that about 3.5 tons of manganese slag are formed during the production of a single ton of mining manganese [3,4]. Large quantities of MS may be discarded or piled up on roads, farmland, or open spaces and thus occupy extensive areas of land. MS also flows into rivers and lakes with the washing of rainwater, causing serious pollution [5,6]. If a more effective utilization of MS could be achieved, potentially high social and economic benefits might result [7]. Therefore, the issue of how best to use manganese slag is an urgent one that needs to be addressed.

Cement is still one of the most widely used building materials today. However, cement production results in large amounts of CO_2_ emissions, and a lot of energy is consumed during its production process. Each year, 4.312 billion tons of cement are produced, resulting in 2.13 billion tons of CO_2_ emissions [8]. To save energy and reduce CO_2_ emissions, some mineral additions have been applied as cement replacements [9]. However, the cost of producing such mineral additions is high. For example, silica fume and blast-furnace slag powder cost USD 145 and USD 85, respectively [10]. Because of this, some solid wastes such as waste fly ash, stove ash, and steel slag have also been used as substitutes for cement. Manganese slag with certain amounts of active substances may also be used for the manufacturing of cement matrix [11]. MS contains toxic heavy metal substances, but these may be leached if the cement materials possess high numbers of pores.

High-performance concrete (HPC) is a type of concrete characterized by high strength, durability, workability, and volume stability. Consequently, HPC may serve as an excellent material for the solidification of MS [12,13]. However, large amounts of cementitious materials are typically required for the preparation of HPC, and such materials are expensive. Therefore, other cementitious materials should be considered for incorporation into HPC. In light of this, some research on solid-waste cementitious material for HPC is of particular interest. In recent studies, fly ash, steel slag, and furnace ash have all been shown to improve the mechanical performance and the durability of HPC [14,15]. In one study, the addition of fly ash was found to increase flexural and compressive strengths by 0~26.1% and 0~33.7%, respectively. In similar works, additions of steel slag and furnace ash were found to increase flexural strength by 0~23.1% and 0~31.3%, respectively [16,17], and compressive strength by 0~18.6% and 0~12.3%, respectively [18,19]. Researchers have also shown that fly ash, steel slag, and furnace ash can all decrease the effects of chloride ions on RDME [20,21,22]. 

HPC is frequently affected by salt erosion when it is applied in coastal environments [23]. Therefore, it is important to identify any changes in the mechanical properties of HPC which result from exposure to salt environments [24]. However, to the best of our knowledge, there has been no research on the performance of HPC with manganese slag under the action of salt.

In the present study, we sought to investigate the effects of adding MS (with mass ratios of 0~40%) and reinforced fibers (steel and basalt fibers, with volume ratios ranging from 0% to 2%) on the mechanical strengths (flexural and compressive strengths) and the microproperties of HPC under conditions of salt erosion. Scanning electron microscope energy-dispersive spectrometer (SEM-EDS) spectra and X-ray diffraction (XRD) curves were obtained to analyze the composition and phases of elements. The findings reported here may contribute to the development of new types of cementitious materials for HPC and new solidification methods for manganese slag. Our results may also serve as a reference for the application of MS-HPC in coastal salt environments in the future.

## 2. Materials and Methods

**Figure 1:**
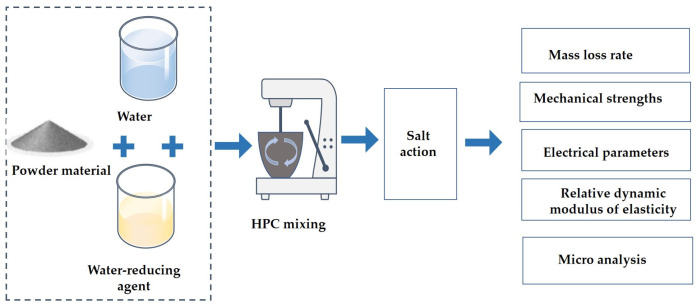
The flowchart of the experimental process.

Figure 1 shows a flowchart of the work carried out in the present study. It shows the preparation of materials, measurement of various performance indicators, and the testing of specimens after salt erosion. In the following text, a more systematic description and analysis of the work is presented.

### 2.1. Raw Materials

**Figure 2:**
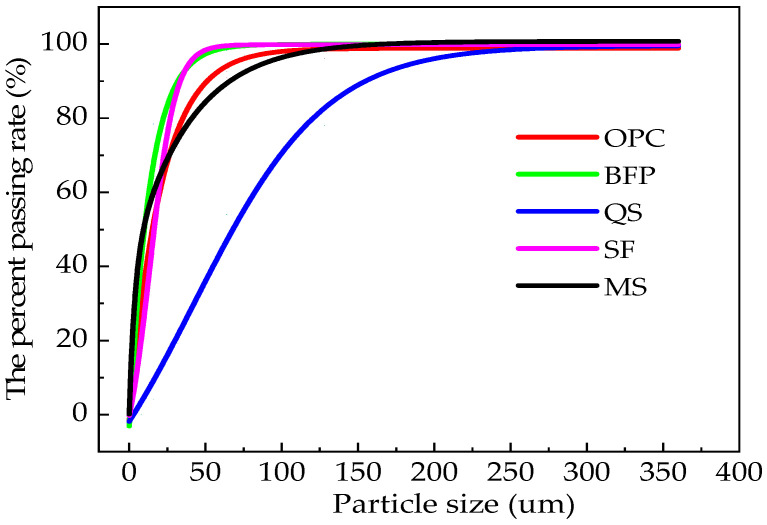
The particle size distribution curves of the raw materials [25].

The Ordinary Portland Cement (OPC) used in this study was sourced from Jiangsu Changlong Cement Manufacturing Co., Ltd., Xuzhou, China. It had a density of 3.01 g/cm^3^, an initial setting time of 117 min, and a final setting time of 226 min. The strength class of the OPC was 45 MPa. We also used a type of mineral admixture known as ultra-fine silica fume (SF), provided by Nanjing Hongqian Environmental Protection Engineering Co., Ltd., Nanjing, China. This silica fume had a density of 2.31 g/cm^3^, a specific surface area of 14.8 m^2^/g, and a SiO_2_ content of higher than 97.4%. The additive material used in this study included manganese slag (MS) obtained from Beijing Baolaier Technology Co., Ltd., Beijing, China. MS was a byproduct produced by electrolytic manganese ore. The blast-furnace slag powder (BFP) was provided by Lingshou County Qiangdong Mineral Products Processing Factory, Shijiazhuan, China. The BFP shows the density and the specific surface area of 2.91 g/cm^3^ and 436.2 m^2^/g, respectively. Quartz sand (QS), sourced from the Lingshou County Quanfeng Mineral Products Processing Factory in Lingshou, China, was used as the aggregate. The particle size ranges for the quartz sand were 1 mm~0.5 mm, 0.5 mm~0.1 mm, and 0.1 mm~0.01 mm. To adjust the fluidity of the fresh high-performance concrete (HPC), we used a TD-JSS1 polycarboxylic acid high-range water-reducing agent (HRWR); this was provided by Shanxi Kebang Building Materials Co., Ltd., Yuncheng, China. The setting times, densities, and physical properties of the raw materials were readily provided by the suppliers. Table 1 and Table 2 show the cumulative retained fractional pass rates and chemical compositions, respectively, of the raw materials. Particle size distribution curves for the raw materials are shown in Figure 2, which is obtained from Xu’s paper [25]. Finally, the basalt fibers used in this study were manufactured by Shandong Taicheng Fiber Co., Ltd., Taian, China. The average lengths and diameters of the basalt fibers were 6 mm and 0.025 mm, respectively, and their corresponding density was 2.631 g/cm^3^.

### 2.2. Specimen Preparations

Table 3 presents the mixing ratios for the HPC. The mass ratios of MS were 0%, 10%, 20%, 30%, and 40% (by total mass of OPC, MS, and SF). The volume ratio of basalt fibers ranged from 0% to 2.0% (with a MS mass ratio of 20%). The mixing proportions were obtained from previously published works, in line with the maximum density theory [26]. The HPC samples were prepared using the following procedure. First, the powder binder materials were loaded into a JJ-5 planetary cement mortar mixer located at the Xianxian Longhui Highway and Railway Test Instrument Factory in Xian, China. They were mixed at a stirring speed of 140 rpm for 30 s. Subsequently, quartz sand was added to the materials, and stirring at 285 rpm was allowed to continue for a further 90 s. Finally, a mixture of water and a water-reducing agent was introduced to the blend, and the mixture was stirred for 120 s at a speed of 285 rpm. Throughout the HPC mixing process, fibers were evenly sprinkled into the mixer. The HPC was manufactured according to the Chinese standard GB/T 2419-2005 [27].

### 2.3. Experimental Methodology

**Figure 3:**
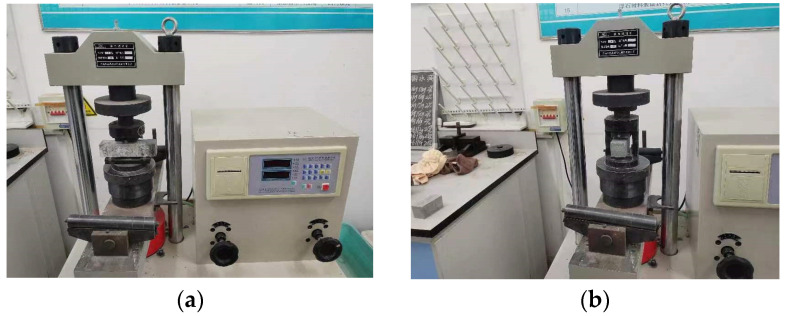
The mechanical strengths of HPC. (**a**) The flexural strength; (**b**) The compressive strength.

To measure mechanical strengths, a fully automatic integrated bending-testing machine was utilized, with loading rates of 0.05 kN/s and 2.4 kN/s to measure flexural and compressive strengths, respectively. Tests of mechanical strength were conducted on specimens with dimensions of 40 mm × 40 mm × 160 mm. The bending strength of the 40 mm × 40 mm × 160 mm specimen was taken as the flexural strength. The two halves of each broken specimen were then used for the measurement of the compressive strength. Therefore, three specimens were used for measuring flexural strengths and six specimens were used for determining compressive strengths. All specimens were cured in a standard curing environment (temperature of 20 ± 2 °C and relative humidity of higher than 95%) for 28 days. The process used for measuring the HPC’s mechanical strengths is shown in Figure 3. The mechanical strengths were tested with reference to the Chinese standard GB/T17671-2005 [28].

### 2.4. The NaCl F-C Action

**Figure 4:**
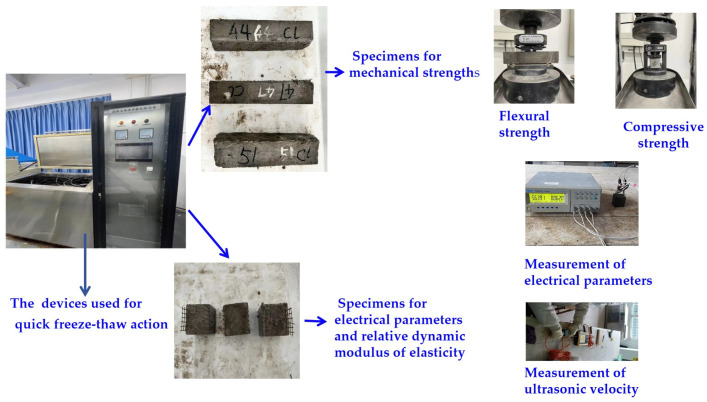
The experiment of quick freeze–thaw action.

Specimens were immersed in a 3% NasCl solution for four days before being transferred to a DR-10 fully automatic rapid freeze–thaw test box manufactured by Tianjin Tianyu Experimental Equipment Co., Ltd., Tianjin, China. The specimens were then placed in sealed stainless-steel freeze–thaw boxes filled with NaCl solutions of the same concentration. The temperature in the F-C box was in a range of −15 °C to 8 °C. The freezing time for each NaCl freeze–thaw cycle (N-FC) was 2~3 h, while the thawing time was 1~2 h. These parameters were obtained after 50 repetitions of the N-FC process. Figure 4 illustrates the N-FC process.

### 2.5. The NaCl and Na_2_SO_4_ D-A Actions

**Figure 5:**
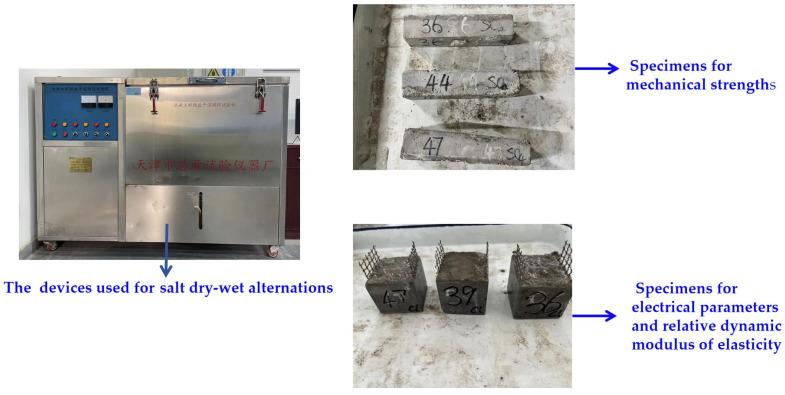
The experiment of Na_2_SO_4_ DA action.

For the dry–wet alternation (N-DA) experiments, the same procedure as that used for NaCl F-C was used to immerse the specimens in NaCl. After soaking, the specimens were transferred to a concrete corrosion resistance dry–wet cycle tester supplied by Zhejiang Miaoda Instrument Manufacturing Co., Ltd., Shaoxing, China. During each D-A cycle, a specimen was placed in a solution of NaCl or Na_2_SO_4_ for 8 h; after this, the specimen surface was dried with a rag. The specimen was then dried at 80 °C for 36 h, followed by a 2-h cooling period. Figure 5 depicts the process of Na_2_SO_4_ D-A (NS-DA). The mechanical strengths were measured per 100 N-FC, 10 N-DA, and 10 NS-DA, using the process described in Section 2.3 above.

### 2.6. The Measurement of Mass Loss Rate

The masses of specimens were measured by immersing them in NaCl for four days, wiping their surfaces, and then weighing them using a BWS-SNR electronic scale purchased from Heng Edge Electronic Technology Co., Ltd., Foshan, China. The scale had a measurement range of 0~3 kg and a minimum measurement of 0.1 g. The mass loss rate (*MLR*) was calculated using Equation (1), as follows:

(1) $$MLR=\frac{m_{t}-m_{1}}{m_{1}}$$

where *m*_1_ represents the mass of samples after 4 days of immersion in 3% NaCl solution, and *m_t_* represents the mass of samples after 50 F-C or 10 D-A actions.

### 2.7. The Measurement of Relative Dynamic Modulus of Elasticity

**Figure 6:**
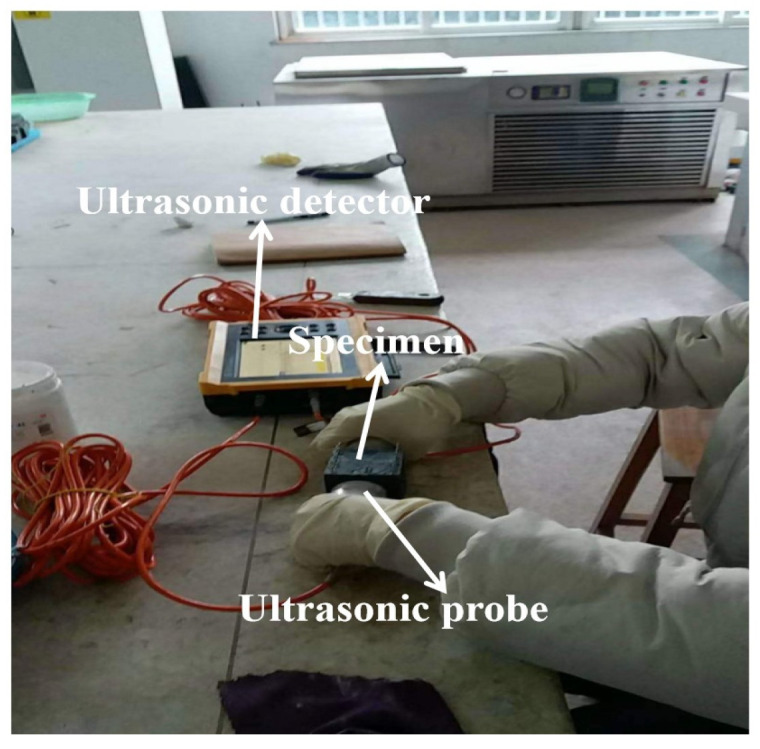
The ultrasonic velocity test process.

A CSM900C digital ultrasonic flaw detector produced by Sanmukeyi Instrument Testing Technology Co., Ltd., Jinan, China, was used to obtain the relative dynamic modulus of elasticity (*RDME*). A transmit-and-receive ultrasonic head was attached firmly to a central position on each specimen. Probes and specimens were matched using petroleum. Ultrasonic velocities were measured as shown in Figure 6, and RDME was calculated using Equation (2), as follows:

(2) $$RDME=\;(\frac{v_{t}}{v_{1}}{)}^{2}$$

where *v_1_* and *v_t_* are the ultrasonic velocities of the samples after periods of salt action of 0 and *t*, respectively. MLR and RDME were measured using samples with dimensions of 100 × 100 × 100 mm^3^. Figure 4 shows a flowchart of the experimental procedure. The measuring method was according to the standard UNE-CEN/TS 12390-2008 [29].

### 2.8. The Measurement of Leached Toxic Heavy Metal Substances

Specimens with a size of 100 × 100 × 100 mm^3^ were used to determine the presence of any leached toxic heavy metal substances during a 6-month period of immersion in deionized water. Concentrations of dissolved chromium (Cr) and zinc (Zn) were measured monthly using an inductively coupled plasma emission spectrometer supplied by Shanghai Meishan Instrument Co., Ltd., of Shanghai, China. In the present study, three specimens were measured in each test, and an average value was used for each experiment.

### 2.9. The Energy-Dispersive X-ray Spectroscopy and XRD Experiment

To obtain scanning electron microscope (SEM) images and energy-dispersive X-ray spectroscopy (EDS) results, an SU3800 scanning electron microscope, purchased from Shanghai Weihan Optoelectronic Technology Co., Ltd. of Shanghai, China, was used. Sample cores were extracted, dried in an oven at 105 °C for two days, subjected to vacuum gold spraying, and then analyzed using the SU3800 scanning electron microscope. SEM and EDS measurements were then obtained.

Samples were also ground into powder, which was used for XRD diffraction measurements using a Bruker JV-DX X-ray diffractometer provided by Shanghai Erdi Instrument Technology Co., Ltd. of Shanghai, China. The samples were characterized by X-ray diffraction (XRD) analysis (Empyrean XRD, PANalytical, Almelo, The Netherlands) with a monochromator using Cu Kα radiation (1.5406 Å). Spectra were acquired in a range from 10° to 70° at 40 kV with a scanning speed of 8° min^−1^.

Some of the powder was then used for thermogravimetric analysis using a TGA thermogravimetric analyzer supplied by Shanghai Farui Instrument Technology Co., Ltd. of Shanghai, China.

The method of thermal analysis was as follows. First, a sample with a mass of 100 mg was weighed after being passed through a 0.1 mm sieve. The sample was then placed on the thermal balance of an STA6000 thermogravimetric analyzer (Shanghai Zhunquan Instrument Equipment Co., Ltd., Shanghai, China). The temperature in the thermogravimetric analyzer ranged from 30 °C to 900 °C, with a heating rate of 10 °C/min and a nitrogen gas flow rate of 150 mL/min to reduce the influence of carbonation on the sample.

## 3. Results and Discussion

### 3.1. The MLR of HPC

**Figure 7:**
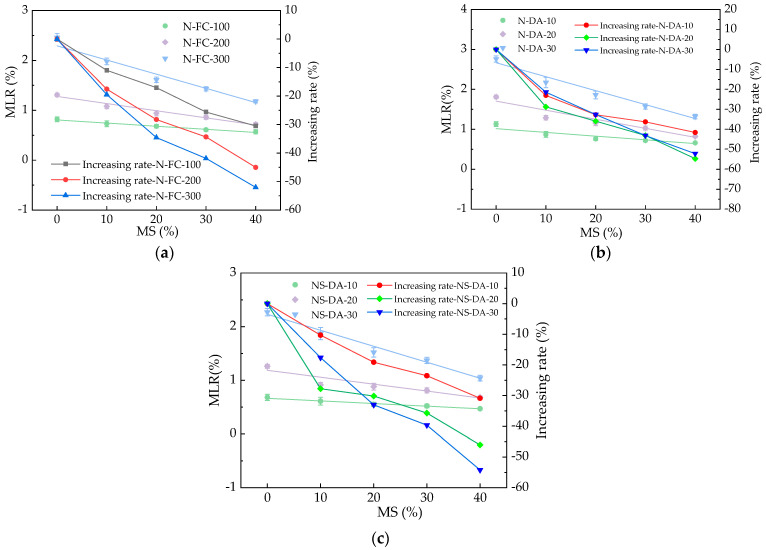
The MLR of HPC under salt action. (**a**) The MLR of HPC during NaCl freeze–thaw cycles. (**b**) The MLR of HPC during NaCl dry–wet alternations. (**c**) The MLR of HPC during Na_2_SO_4_ dry–wet alternations.

The MLR of HPC is shown in Figure 7. The continuous lines on the graph represent the fitting curves of the experimental results. The N-FC, N-DA, and NS-DA were applied to the specimens. An increasing trend of mass was noted during the periods of N-FC, N-DA, and NS-DA, which was due to the erosion of N-FC, N-DA, and NS-DA [30]. Once, the N-FC acts on the HPC specimens, the NaCl crystals will continuously dissolve and precipitate, which results in continuous generation and dissipation of stress [31]. The round-trip stress induces the propagation and increase of internal cracks in HPC. Consequently, the mass decreases with the number of N-FC, leading to an increase in the MLR of HPC. Moreover, the freezing and thawing stress from N-FC can cause surface detachment of HPC, resulting in the increased mass. The NaCl penetrated into the pores of HPC when HPC was immersed in NaCl solution [32]. When the HPC was dried, the NaCl crystals precipitated. The wet and dry process led to NaCl infiltration, precipitation, and dissolution cycling; therefore, the crack and spalling of specimens occurred, resulting in the reduction of mass [33]. During the sulfate attack process, concrete was not only affected by the expansion and damage of corrosion products such as ettringite and gypsum but also by the crystallization and expansion of Na_2_SO_4_·10H_2_O during the dry–wet cycle, causing repeated and continuous accumulation of concrete damage, accelerating the rate of corrosion and deterioration of concrete, and gradually reducing the mass [34]. Moreover, as observed in Figure 7, the MLR decreased with the mass ratio of MS, due to the filling effect and the pozzolanic effect [35]. The decreasing rates of HPC’s MLR were 0~52.1%, 0~56.3%, and 0~54.8%. When the microcracks appeared inside the HPC, a large amount of water continued to penetrate the cracks, accelerating the secondary hydration reaction of MS, and the generated hydration products blocked the microcracks [36]. Therefore, under the action of N-FC, N-DA, and NS-DA, the mass of HPC increases. The error bars’ values were lower than 8.5% of the real values of MLR, indicating the experimental correctness. The MLR after 30 N-DA was the highest, and the MLR after 30 NS-DA was the lowest. Table 4 shows the fitting equations of the relationships between the MLR and MS’s mass ratios. As observed in Table 4, the equations were linear functions, and the fitting degrees of the fitting equations were higher than or equal to 0.92, ensuring the reasonability of fitting functions. Compared with the UHPC with waste fly ash and secondary aluminum ash, the HPC with MS showed a 0~0.71% lower MLR.

### 3.2. The RDME of HPC

**Figure 8:**
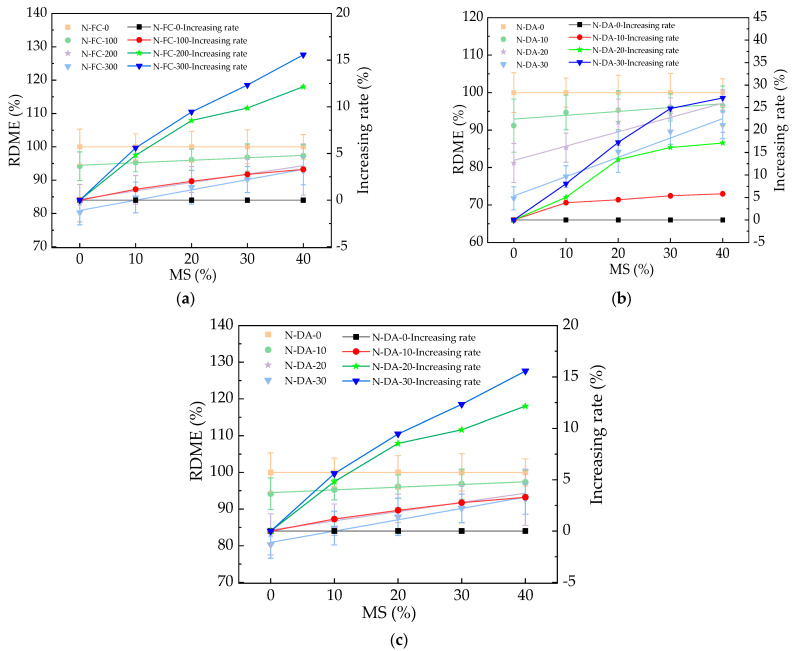
The RDME of HPC under salt action. (**a**) The RDME of HPC during NaCl freeze–thaw cycles. (**b**) The RDME of HPC during NaCl dry–wet alternations. (**c**)The RDME of HPC during Na_2_SO_4_ dry–wet alternations.

The RDME of HPC after different cycles of N-FC, N-DA, and NS-DA are provided in Figure 8. The RDME of HPC decreased with the effect of N-FC, N-DA, and NS-DA. This was attributed to the width and the number of internal cracks in HPC by the salt action, which slowed down the propagation of sound waves in HPC [37]. Consequently, the RDME of HPC was decreased by the effects of N-FC, N-DA, and NS-DA. Meanwhile, the RDME of HPC was increased by adding the MS with the increasing rates of 0~15.7%, 0~27.1%, and 0~13.7%, respectively, under the actions of N-FC, N-DA, and NS-DA, which was ascribed to the microaggregate effect and the pozzolanic effect just in the analysis of Section 3.1 [38]. The error bars’ values were lower than 7.9% of the real MRL values, ensuring the experimental correctness. HPC showed the highest RDME after 30 NS-DW. Meanwhile, the RDME of HPC was the lowest after 30 N-DA. Table 5 demonstrates the fitting equations of the relationships between the RDME and MS’s mass ratios. As depicted in Table 5, the equations are linear functions, and the fitting degrees of the fitting equations are higher than or equal to 0.90, ensuring the reasonability of fitting functions. The RDME of HPC was 0~7.1% higher than the HPC with waste fly ash and secondary aluminum ash.

### 3.3. The Mechanical Strengths of HPC

**Figure 9:**
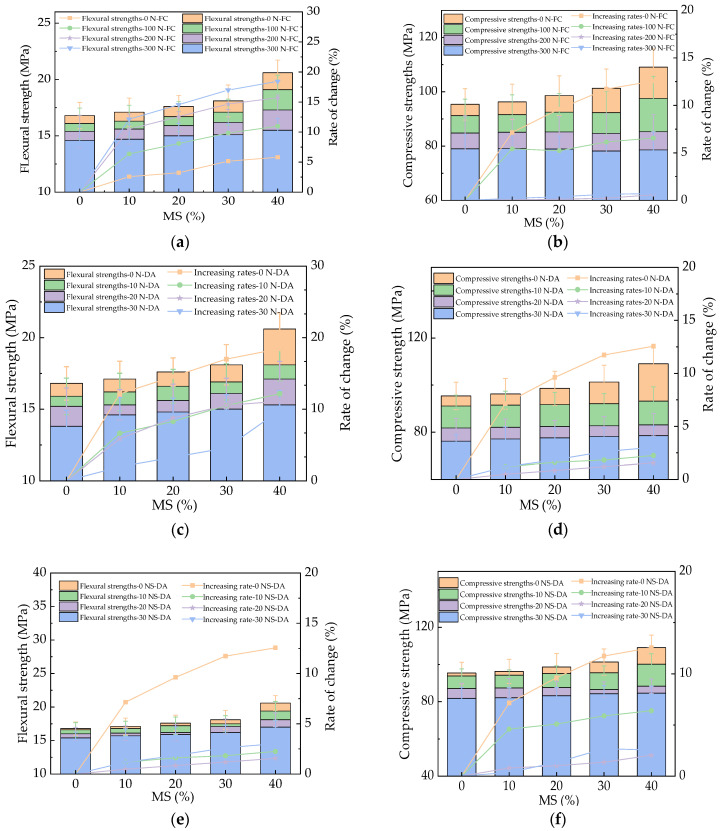
The mechanical strengths of HPC under salt erosion. (**a**) HPC flexural strength during NaCl freeze–thaw cycles. (**b**) HPC compressive strength during NaCl freeze–thaw cycles. (**c**) HPC flexural strength during NaCl dry–wet alternations. (**d**) HPC compressive strength during NaCl dry–wet alternations. (**e**) HPC flexural strength during Na_2_SO_4_ dry–wet alternations. (**f**) HPC compressive strength during Na_2_SO_4_ dry–wet alternations.

Figure 9 provides the flexural and compressive strengths of HPC with MS during the N-FC, N-DA, and NS-DA actions. The mechanical strengths of HPC were decreased by N-FC, N-DA, and NS-DA actions. This could be explained by the increased number and length of cracks in HPC, leading to decreased mechanical strengths [39]. As depicted in Figure 9, after salt action, the flexural strengths were increased by the added MS with varying rates of 0~17.3%, 0~18.6%, and 0~17.1% under the N-FC, N-DA, and NS-DA actions, respectively. Meanwhile, the corresponding compressive strengths were increased by the rates of 0~11.7%, 0~12.63%, and 0~11.3%. The microaggregate effect and the pozzolanic effect were improved by adding the MS, which decreased the internal crack propagation, resulting in a decline in mechanical strength loss [40]. The mechanical strengths of HPC after NS-DA were the highest, while the corresponding mechanical strengths of HPC after N-DA were the lowest. The error bars’ values were lower than 8.2% of the real values of mechanical strengths, indicating the experimental accuracy. Compared with the HPC with fly ash and secondary aluminum ash, the flexural and compressive strengths of HPC were 0~11.3% and 0~17.2% higher [23].

### 3.4. The Influence of Basalt Fibers

**Figure 10:**
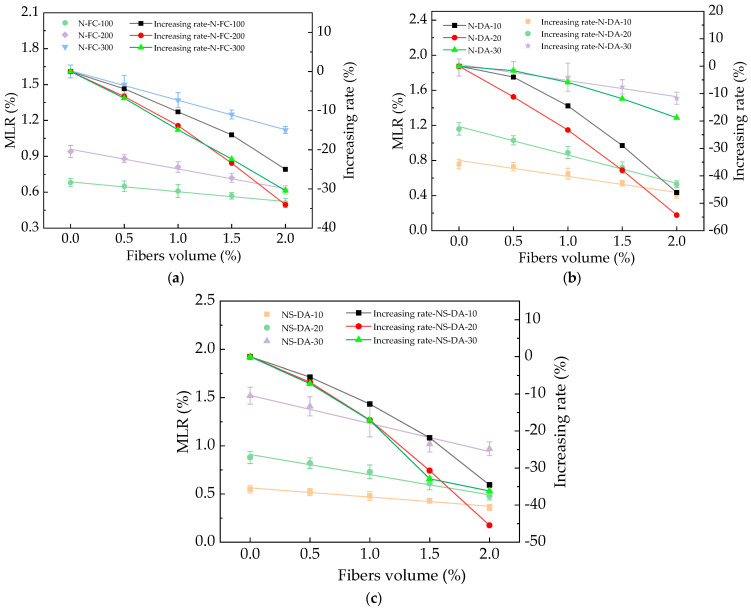
The MLR of HPC with fibers under salt action. (**a**) The MLR of HPC with fibers during NaCl freeze–thaw cycles. (**b**) The MLR of HPC with fibers during NaCl dry–wet alternations. (**c**) The MLR of HPC with fibers during Na_2_SO_4_ dry–wet alternations.

The MLR of HPC after different numbers of N-FC, N-DA, and NS-DA actions are shown in Figure 10. As shown in Figure 10, the MLR increased with the increasing numbers of N-FC, N-DA, and NS-DA and decreased with the added basalt fibers with the decreasing rates of 0~34.8%, 0~55.1%, and 0~45.3%, respectively. The basalt fibers showed decreasing effects on the increasing rates of MLR by the N-FC, N-DA, and NS-DA. This was because the added basalt fibers can limit cracks in HPC to achieve the goal of reducing surface peeling and reducing the MLR [41]. After salt action, the MLR of HPC with basalt fibers was the highest after suffering 40 N-DA. Meanwhile, HPC with basalt fibers showed the lowest MLR after 40 NS-DA. The error bars’ values were lower than 9.3% of the real values of the MLR, ensuring the experimental accuracy. The fitting equations of the relationships between the MLR and BF’s volume ratios are illustrated in Table 6. As depicted in Table 6, the equations were linear functions, and the fitting degrees of the fitting equations were higher than or equal to 0.95, which ensured that most of the variance of the tests (>95%) was explained by the linear fit.

**Figure 11:**
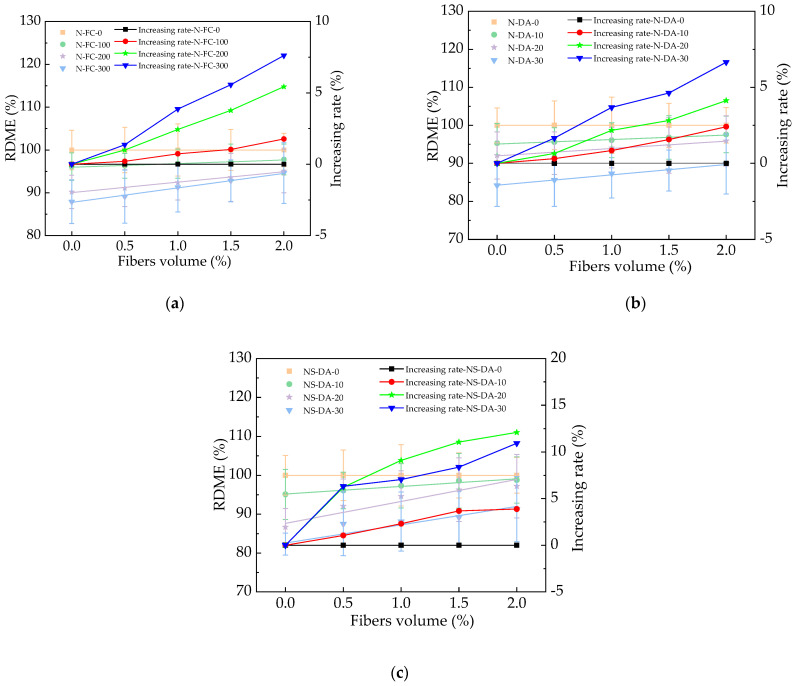
The RDME of HPC with fibers under salt action. (**a**) The RDME of HPC with fibers during NaCl freeze–thaw cycles. (**b**) The RDME of HPC with fibers during NaCl dry–wet alternations. (**c**) The RDME of HPC with fibers during Na_2_SO_4_ dry–wet alternations.

The RDME of HPC with basalt fibers is depicted in Figure 11. As shown in Figure 11, the HPC’s RDME decreased after 300 N-FC, 30 N-DA, and 30 NS-DA. After the basalt fibers were added, the corresponding decreasing rates of RDME by basalt fibers were decreased by 0~7.62%, 0~6.65%, and 0~12.11%, respectively. The reason for this was that the basalt fibers could bridge the cracks in HPC, which limited the propagation of cracks [42]. Therefore, a decrease in the speed of sound propagation in HPC occurred. The RDME of HPC with basalt fibers after 30 NS-DW was the highest, while HPC with basalt fibers showed the lowest RDME after 30 N-DW. The corresponding error bars’ values were lower than 9.1% of the real values of the HPC’s RDME, which indicated the experimental exactitude. The fitting equations of the relationships between the RDME and BF’s volume ratios are illustrated in Table 7. As depicted in Table 7, the equations were linear functions, and the fitting degrees of the fitting equations were higher than or equal to 0.91, which ensured the reasonability of fitting functions.

**Figure 12:**
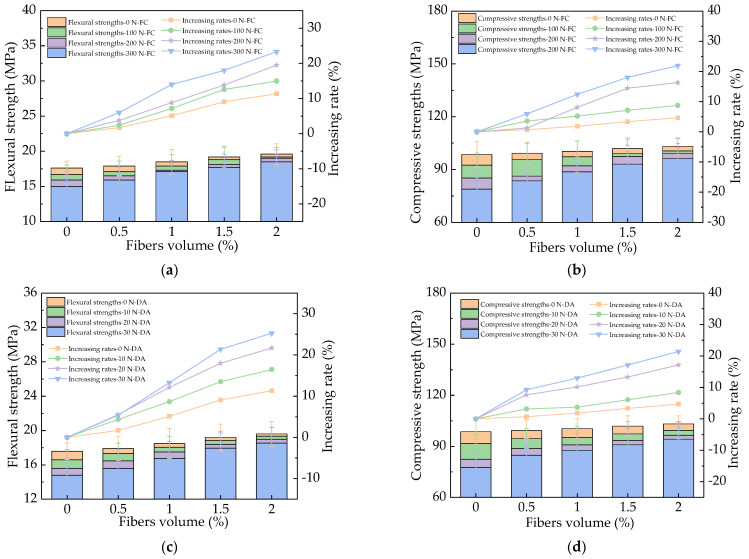

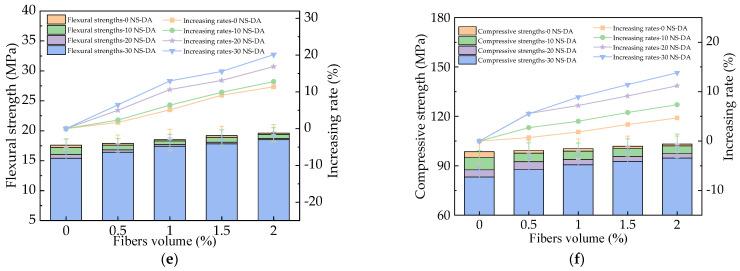
The mechanical strengths of HPC with fibers under salt erosion. (**a**) HPC with fibers flexural strength during NaCl freeze–thaw cycles. (**b**) HPC with fibers compressive strength during NaCl freeze–thaw cycles. (**c**) HPC with fibers flexural strength during NaCl dry–wet alternations. (**d**) HPC with fibers compressive strength during NaCl dry–wet alternations. (**e**) HPC with fibers flexural strength during Na_2_SO_4_ dry–wet alternations. (**f**) HPC with fibers flexural strength during Na_2_SO_4_ dry–wet alternations.

The mechanical strengths of HPC with basalt fibers are provided in Figure 12. As illustrated in Figure 12, the mechanical strengths obviously decreased after the actions of N-FC, N-DA, and NS-DA due to the increased cracking by salt action [43]. After the basalt fibers were added, the increasing rates of flexural strength were 0~22.1%, 0~23.6%, and 0~20.4% after 300 N-FC, 30 N-DA, and 30 NS-DA. The corresponding compressive strengths’ increasing rates were 0~21.3%, 0~20.2%, and 0~13.6% after salt action. The corresponding error bars’ values were lower than 9.1% of the real values of the HPC’s mechanical strengths, indicating the experimental accuracy.

### 3.5. The Microscopical Properties

**Figure 13:**
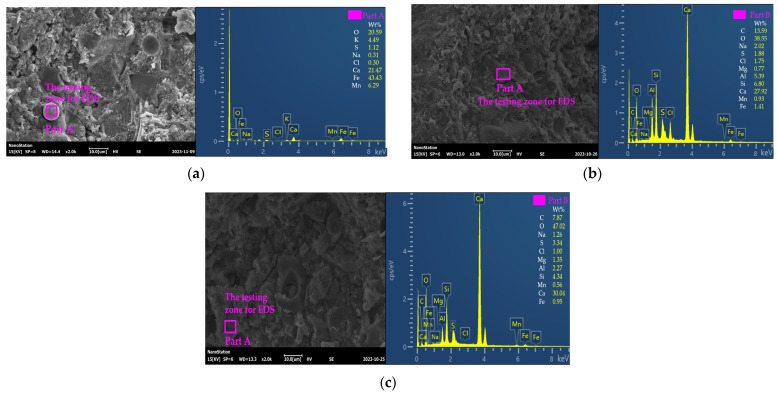
The SEM-EDS of HPC with MS under salt erosion. (**a**) The SEM-EDS photos of specimens before salt action. (**b**) The SEM-EDS photos of specimens after 30 NaCl dry–wet alternations. (**c**) The SEM-EDS photos of specimens after 30 Na_2_SO_4_ dry–wet alternations.

Figure 13 provides the SEM-EDS of HPC with MS after salt action. As depicted in Figure 13, the hydration products consisted of compact hydration products, flocculent hydration products, and needle hydration products. As shown in Figure 13, more flocculent hydration products were found after the NS-DA action. The element of Cl was increased after N-DA actions. The cracked hydration products were increased by adding NaCl actions. The flocculent hydration products are used to describe the morphology of the new hydration product induced by salt action. Compared with the ordinary compact structure, the flocculent hydration products identified by SEM can degrade the mechanical performance of HPC. The MS demonstrated the increasing effects on flocculent hydration products and the elements of Mn, Mg, and Fe.

**Figure 14:**
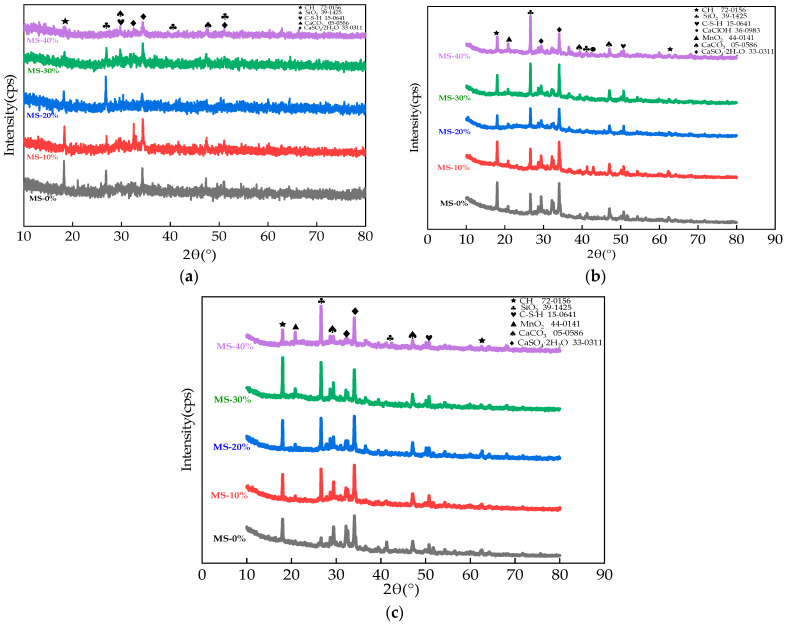
The XRD curves of the HPC. (**a**) The XRD curves of specimens before salt action. (**b**) The XRD curves of specimens after 30 NaCl dry–wet alternations. (**c**) The XRD curves of specimens after 30 Na_2_SO_4_ dry–wet alternations.

The XRD curves of HPC with MS are shown in Figure 14. As shown in Figure 14, the crystals of SiO_2_, Ca(OH)_2_ (CH), CaSO_4_·2H_2_O, calcium silicate hydrate (C-S-H), and CaCO_3_ were discovered in all specimens. The CaClOH crystals were observed after the NaCl dry–wet actions. This was attributed to the fact that the actions of the dry–wet alternations could increase the hydration between the cement and the NaCl solution, thus forming the chloride complex salt. When the 30 Na_2_SO_4_ dry–wet alternations actions were finished, the diffraction peaks of Fe_2_SO_3_ were increased by the increasing dosages of MS, since the Fe content was increased by the added MS [44]. When the dry–wet alternations of Na_2_SO_4_ were applied to the specimens (the iron), the diffraction peaks of Fe_2_SO_3_ crystals increased. Moreover, the CaSO_4_ crystals’ diffraction peaks were increased by the dry–wet alternations with Na_2_SO_4_ solution. This could be explained by the reaction of the Ca(OH)_2_ and the Na_2_SO_4_, forming the CaSO_4_ [45].

**Figure 15:**
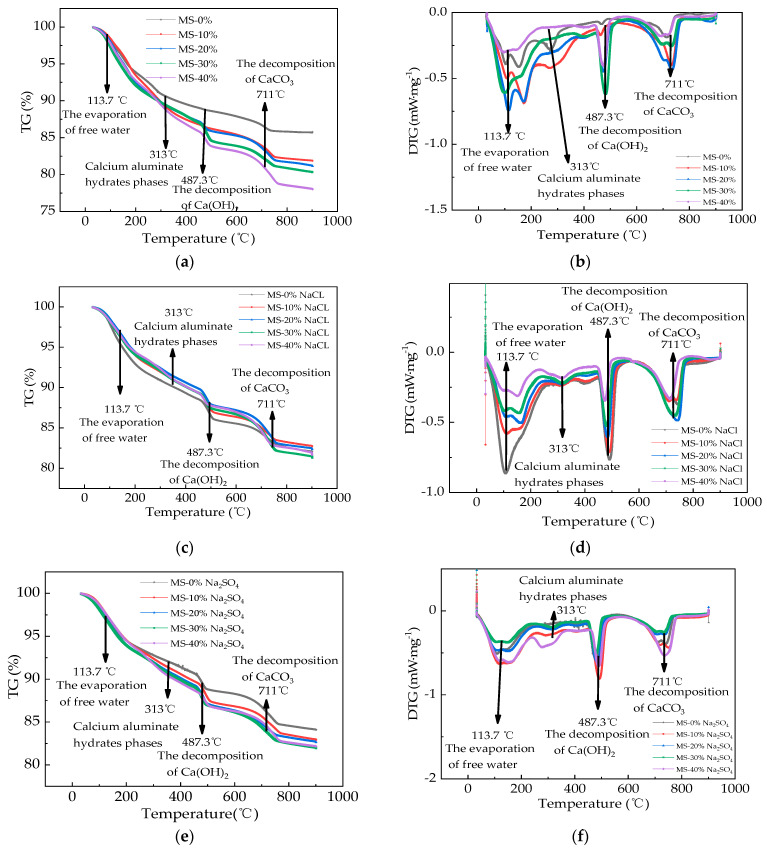
The thermal analysis curves of the HPC. (**a**) The TG curves of specimens before salt action. (**b**) The DTG curves of specimens before salt action. (**c**) The TG curves of specimens after 30 NaCl dry–wet alternations. (**d**) The DTG curves of specimens after 30 NaCl dry–wet alternations. (**e**) The TG curves of specimens after 30 Na_2_SO_4_ dry–wet alternations. (**f**) The DTG curves of specimens after 30 Na_2_SO_4_ dry–wet alternations.

The TG curves of HPC with MS are provided in Figure 15. In Figure 15, the curves can be divided into four parts. In stage one, the temperature varied from 33 °C to 113.7 °C (the first peak shown in Figure 15b,d,f), and the TG showed a declining trend ranging from 100% to 94.3%. This could be explained by the evaporation of free water in pore solution [46]. In the second stage, the TG values decreased from 94.3% to 86.7% with the temperature ranging from 113.7 °C to 487.3 °C due to the decomposition of calcium silicate hydrate (C-S-H) and calcium aluminate hydrates phases [47]. The second peak (313 °C shown in Figure 15b,d,f) indicated the calcium aluminate hydrates phases. In the third stage, the TG values’ range was 86.7~82.8% with the temperature ranging from 487.3 °C to 711 °C, which was ascribed to the decomposed Ca(OH)_2_ hydration products. In the fourth stage, the TG values varied from 82.8% to 80.7% with the temperature ranging from 711 °C to 900 °C, which was ascribed to the CaCO_3_’s decomposition. The addition of MS could increase the degree of decline in the TG values. This could be explained by the improved pozzolanic effect of MS. Therefore, the TG values were decreased by the added MS [48]. When the salt actions were exerted on the specimens, the decline in the TG values decreased. This was attributed to the infiltration and decomposition of salts [49]. The TG values showed the least decline after the 30 N-DW.

## 4. Conclusions

In this study, changes in the mechanical strength of HPC with added MS during exposure to N-FC, N-DW, and NS-DW were investigated experimentally. The study conclusions may be summarized as follows:

With additions of MS ranging from 0% to 40%, the MLR of HPC was found to decrease in ranges of 0% to 52.1%, 0% to 56.3%, and 0% to 54.8% during N-FC, N-DW, and NS-DW actions, respectively. Furthermore, for these same salt actions, the incorporation of BFs led to MLR reduction rates in ranges of 0% to 34.8%, 0% to 55.1%, and 0% to 45.3%, respectively. The addition of MS and BFs also had the effect of increasing the RDME. When MS was added, the RDME increased in ranges of 0~15.7%, 0~27.1%, and 0~13.7% after N-FC, N-DW, and NS-DW actions, respectively. When BFs were added, the corresponding increases in RDME values for the same actions were in the ranges of 0~34.8%, 0~55.1%, and 0~45.3%, respectively.

The mechanical strengths of HPC were, therefore, improved by the addition of MS and BFs. When MS was added, flexural strength increased by 0% to 17.3%, 0% to 18.6%, and 0% to 17.1% under N-FC, N-DW, and NS-DW actions, respectively. For the same actions, the corresponding increases in compressive strength were 0~11.7%, 0~12.63%, and 0~11.3%, respectively. Similarly, when BFs were added, the flexural strength increased by 0~22.1%, 0~23.6%, and 0~20.4% under N-FC, N-DW, and NS-DW actions, respectively. For the same cycles, the corresponding increases in compressive actions were 0~21.3%, 0~20.2%, and 0~13.6%, respectively.

The addition of MS resulted in an increased presence of flocculent hydration products and the elements Mn, Mg, and Fe. In the XRD curves, CaClOH and CaSO_4_ ·2H_2_O crystals were found after the N-DW and NS-DW actions. The variation in TG values could be increased by the addition of MS.

In conclusion, in the present work, we found that the addition of MS and BFs proved effective in enhancing the resistance of HPC to salt erosion. This finding may be attributed to the volcanic ash effect in the case of MS, and the bridging effect in the case of BFs.

## Figures and Tables

**Table 1:** The cumulative retained fractional pass rates (%).

Types	Particle Size/μm						
	0.3	0.6	1	4	8	64	360
OPC	0.13	0.36	3.08	14.79	29.13	92.62	100
SF	32.03	59.02	82.98	99.9	99.8	100	100
QS	0	0	0	0	0.035	23.94	100
MS	0.05	0.32	1.33	8.72	23.52	98.43	100
BFP	0.04	0.12	3.3	19.4	35.2	98.2	100

**Table 2:** Chemical composition (wt%).

Types	SiO_2_	Al_2_O_3_	Fe_x_O_y_	MgO	CaO	SO_3_	K_2_O	Mn_2_O	Loss on Ignition
OPC	20.1	5.8	3.1	1.9	62.2	3.0	-	-	3.0
SF	90	0.4	0.5	0.6	0.35	0.3	7.4	-	-
QS	98.3	-	1.5	-	-	-	-	-	-
MS	34.22	7.32	12.03	1.91	13.92	0.08	-	30.52	-
BFP	34	14.9	0.5	9.8	36.9	0.3	3.6	-	-

**Table 3:** The mixing proportions of HPC with MS and BFs (kg/m^3^).

Samples	Water	OPC	MS	SF	BFP	QS	BFs	HRWR
MS-0%	240	658	0	183	111.1	962	0	16
MS-10%	240	567	91	183	111.1	962	0	16
MS-20%	240	477	181	183	111.1	962	0	16
MS-30%	240	383	274	183	111.1	962	0	16
MS-40%	240	293	365	183	111.1	962	0	16
BFs-0%	240	477	181	183	111.1	962	0	16
BFs-0.5%	240	477	181	183	111.1	962	13	16
BFs-1.0%	240	477	181	183	111.1	962	26	16
BFs-1.5%	240	477	181	183	111.1	962	39	16
BFs-2.0%	240	477	181	183	111.1	962	42	16

**Table 4:** The fitting equations of the relationships between the MLR and MS’s mass ratios.

Equation	Types	*a*	*b*	R^2^
*MLR = a + bMS*	N-FC-100	0.81	−6.29 × 10^−3^	0.98
	N-FC-200	1.29	−1.43 × 10^−2^	0.97
	N-FC-300	2.29	−2.08 × 10^−2^	0.96
	N-DA-10	1.01	−9.34 × 10^−3^	0.92
	N-DA-20	1.71	−2.23 × 10^−2^	0.93
	N-DA-30	2.67	−3.49 × 10^−2^	0.97
	NS-DA-10	0.66	−4.87 × 10^−3^	0.98
	NS-DA-20	1.18	−1.29 × 10^−2^	0.98
	NS-DA-30	2.23	−2.29 × 10^−2^	0.98

**Table 5:** The fitting equations of the relationships between the RDME and MS’s mass ratios.

Equation	Types	*a*	*b*	R^2^
*RDME = a + bMS*	RDME(%)-N-FC-0	100	0	1.00
	RDME(%)-N-FC-100	94.48	0.074	0.97
	RDME(%)-N-FC-200	84.27	0.251	0.91
	RDME(%)-N-FC-300	80.9	0.308	0.98
	RDME(%)-N-DA-0	100	0	1.00
	RDME(%)-N-DA-10	92.92	0.103	0.90
	RDME(%)-N-DA-20	81.89	0.382	0.92
	RDME(%)-N-DA-30	72.4	0.52	0.97
	RDME(%)-NS-DA-0	100	0	1.00
	RDME(%)-NS-DA-10	94.93	0.089	0.98
	RDME(%)-NS-DA-20	88.04	0.258	0.92
	RDME(%)-NS-DA-30	83.73	0.27	0.91

**Table 6:** The fitting equations of the relationships between the MLR and BF’s volume ratios.

Equation	Types	*a*	*b*	R^2^
*MLR = a + bBF*	N-FC-100	0.68	−0.0818	0.98
	N-FC-200	0.96	−0.165	0.99
	N-FC-300	1.62	−0.245	0.99
	N-DA-10	0.80	−0.18	0.95
	N-DA-20	1.19	−0.32	0.99
	N-DA-30	1.89	−0.18	0.96
	NS-DA-10	0.56	−0.095	0.96
	NS-DA-20	0.91	−0.21	0.98
	NS-DA-30	1.52	−0.29	0.96

**Table 7:** The fitting equations of the relationships between the RDME and BF’s volume ratios.

Equation	Types	*a*	*b*	R^2^
*RDME = a + bBF*	N-FC-0	100	0	1.00
	N-FC-100	95.98	0.85	0.96
	N-FC-200	90.04	2.43	0.99
	N-FC-300	87.77	3.38	0.99
	N-DA-0	100	0	1.00
	N-DA-10	95.08	1.18	0.96
	N-DA-20	91.97	1.93	0.98
	N-DA-30	84.26	2.71	0.99
	NS-DA-0	100	0	1.00
	NS-DA-10	95.19	1.96	0.96
	NS-DA-20	87.63	5.65	0.91
	NS-DA-30	82.57	4.71	0.92

## Data Availability

Data are contained within the article.

## Abbreviations

MS	manganese slag
RDME	relative dynamic modulus of elasticity
HPC	high-performance concrete
MS-HPC	manganese slag high-performance concrete
N-FC	NaCl freeze–thaw cycles
N-DA	NaCl dry–wet alternations
NS-DA	Na_2_SO_4_ dry–wet alternations
SEM	scanning electron microscope
EDS	energy-dispersive spectrometer
TG	thermogravimetric analysis
XRD	X-ray diffraction spectroscopy
MLR	mass loss rate
OPC	Ordinary Portland Cement
SF	silica fume
QS	quartz sand
BFP	blast-furnace slag powder
HRWR	high-range water-reducing agent
Cr	chromium
Zn	zinc
C-S-H	calcium silicate hydrate
